# Transient ovarian torsion in a pregnant woman after vitrified-warmed embryo transfer: a case report

**DOI:** 10.5935/1518-0557.20230056

**Published:** 2024

**Authors:** Hoda Sibai, Ahmed Ismail Heraiz, Nadia M. Madkour

**Affiliations:** 1 Assistant professor of Obstetrics and Gynecology, Zagazig University, Zagazig, Egypt; 2 Lecturer of Obstetrics and Gynecology, Zagazig University, Zagazig, Egypt

**Keywords:** ovarian torsion, pregnancy, vitrified-warmed cycles

## Abstract

Assisted reproduction is a risk factor for adnexal torsion due to ovarian hyperstimulation and increased incidence of twin pregnancy. Both risk factors can be eliminated in frozen embryo transfers, but in our case ovarian torsion occurred after the use of an aromatase inhibitor (Femara) in endometrium preparation due to the presence of corpus luteum. Case presentation: G2P1+0 presented at 7 weeks gestation after vitrified-warmed embryo transfer with right loin pain and mild right iliac pain and tenderness. Ultrasound examination revealed transient or incomplete ovarian torsion. The presentation of the case was somewhat misleading and the transient nature of the torsion provided an opportunity for the conservative management of the case. In conclusion, ovarian torsion is still an undesired event, even after single embryo transfers and in vitrified-warmed cycles. Clinical and ultrasound follow-up precluded the need for surgery in our case.

## INTRODUCTION

Adnexal torsion is the fifth most common cause of acute abdominal pain in women during the reproductive period (Amirbekian & Hooley, 2014). Although incidence during pregnancy is uncertain, it is estimated to range from 0.2 to 3% (Huchon & Fauconnier, 2010). It is defined as complete or partial rotation of the adnexa around the ligamentous supports, which contain the vascular pedicle. This results in the interruption of venous reflux and arterial flow, consequently leading to ischemia and necrosis of the affected adnexa.

Pregnancy and assisted reproductive techniques have been implicated as risk factors for adnexal torsion (Houry & Abbott, 2001). Adnexal torsion occurs at any time during pregnancy; however, it often occurs during the first and second trimesters. The symptoms of adnexal torsion are non-specific, and patients with the condition may be misdiagnosed with other causes of acute abdominal pain such as appendicitis and urinary tract infection (Sasaki & Miller, 2014). Clear guidelines for the management of adnexal torsion during pregnancy are lacking, as there is a paucity of studies on this important topic (Wang & Deng, 2020).

## CASE PRESENTATION

G2P1+0, aged 32 years, with a history of primary infertility due to male factor, presented with right renal flank pain and right iliac pain in the 7^th^ week of pregnancy after undergoing a vitrified-warmed embryo transfer. Her first pregnancy was after vitrified-warmed ET. Preparation performed at the time was by hormone replacement therapy (HRT) and two day-5 embryos were transferred. She became pregnant with twins and at 19 weeks of gestation she felt lower abdominal heaviness; ultrasound examination revealed cervical dilatation with bulging of membranes. Emergency cervical cerclage was performed and tocolytic drugs administered, with continuation of pregnancy until 26 weeks, at which time preterm labor started. Two preterm female babies were delivered by Cesarean section and placed on the incubator; unfortunately, the two died, one within 24 hours and the other within six days of birth.

**Figure 1:**
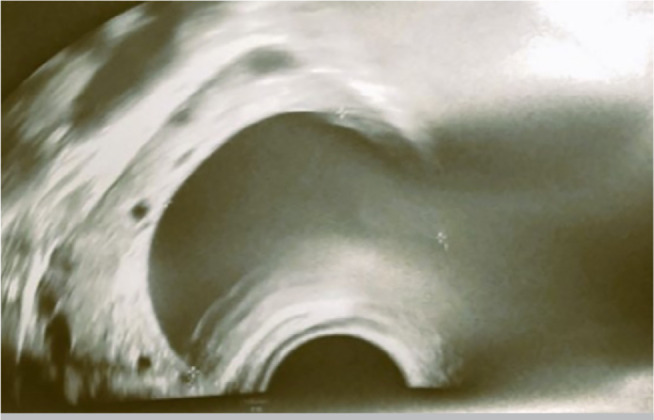
Ultrasound scan showing edema and cyst in the RT ovary.

**Figure 2:**
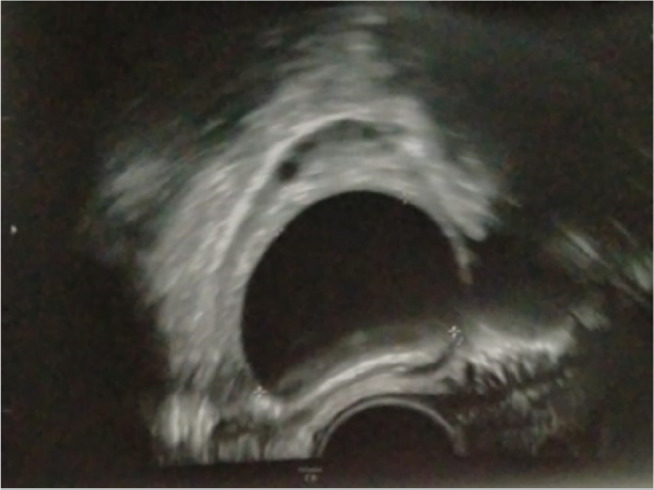
Ultrasound scan of the same ovary six days later showing resolution of edema.

She came in for another FET and was advised to undergo a single embryo transfer. HRT was prescribed for endometrial preparation and a single day-5 embryo was transferred, but she did not get pregnant. Two months later, she underwent another FET after HRT for endometrial preparation, the BhCG test was positive, but it was a case of chemical pregnancy. Two months later, she came in for a third FET, in which preparation of the endometrium was performed with an aromatase inhibitor (Femara) 5 mg daily from day 3 of the cycle for five days. A follow-up visit on day 9 of the cycle found a follicle measuring 16.5 mm on the right ovary and the endometrium measuring 7mm; two days later, the follicle measured 20.5 mm and the endometrium 12.2mm. She was given 10000IU HCG and progesterone was administered for 5 days when a single embryo (4AA) was transferred. Fifteen days later, her BhCG level was 572IU. At 7 weeks of gestation, she developed right loin pain and right iliac pain with altered urinary frequency. On examination, she had a lax abdomen, minimal right iliac tenderness, and maximum pain on the right loin region. Ultrasound revealed a 7-week embryo and a right ovarian cyst measuring 59 x 30mm with clear fluids. Analgesics and antibiotics were given to alleviate the pain, since she had history of urinary stones; right renal flank pain mainly placed UTI as the most probable diagnosis, so urinalysis and urine culture and sensitivity were performed with negative results. Ultrasound scans showed normal findings with no ureteric dilatation or backpressure. Pain decreased 24 hours later, but she still had mild pain in the flank, so another ultrasound was performed which revealed right ovarian edema and minimal vasculature with an ovarian cyst measuring 48 x 44mm, indicative of intermittent or incomplete ovarian torsion (Figures 1). Pain decreased to minimal levels. We decided to follow the patient and perform another ultrasound examination six days later of she did not have pain (Figures 2). Follow-up ultrasound scans showed improved signs and a decrease on the size of the ovarian cyst. Another ultrasound scan was performed at 12 weeks, in which a normal fetus with no suspicion of torsion and an ovarian cyst measuring to 27x16mm were seen. The patient has since been doing well.

## DISCUSSION

Ovarian torsion is an undesired event that may complicate pregnancy. Our patient became pregnant after FET and was administered an aromatase inhibitor (Femara) for endometrial preparation, which resulted in the development of a corpus luteum cyst and ovarian torsion, subsequently. She had atypical signs that, combined with her medical history, were suggestive of urinary tract infection. However, tests excluded this possibility and she was suspected with ovarian torsion, which was later confirmed in ultrasound examination and further described as a transient form of ovarian torsion.

Since she improved from pain, conservative management was chosen and the torsion resolved. Ovarian torsion may at times be challenging to diagnose, particularly in cases in which patients have primarily loin pain, as in our patient. However, ovarian torsion must be considered in the differential diagnosis of patients offered ovarian stimulation for endometrial preparation. Lastly, conservative management of transient or incomplete ovarian torsion can be performed successfully.

## CONCLUSION

The use of an aromatase inhibitor (Femara) in the preparation of the endometrium for vitrified-warmed embryo transfer which results in corpus luteum development might be a risk factor for ovarian torsion. Conservative non-surgical management is an option in cases of transient ovarian torsion.
